# Improved Durability and Sensitivity of Bitterness-Sensing Membrane for Medicines

**DOI:** 10.3390/s17112541

**Published:** 2017-11-04

**Authors:** Xiao Wu, Hideya Onitake, Zhiqin Huang, Takeshi Shiino, Yusuke Tahara, Rui Yatabe, Hidekazu Ikezaki, Kiyoshi Toko

**Affiliations:** 1 Graduate School of Information Science and Electrical Engineering, Kyushu University, 744 Motooka, Nishi-ku, Fukuoka 819-0395, Japan; onihidedlolb@yahoo.co.jp (H.O.); zhiqin1127@126.com (Z.H.); shiino.takeshi.206@s.kyushu-u.ac.jp (T.S.); toko@ed.kyushu-u.ac.jp (K.T.); 2 Research and Development Center for Taste and Odor Sensing, Kyushu University, 744 Motooka, Nishi-ku, Fukuoka 819-0395, Japan; tahara@belab.ed.kyushu-u.ac.jp (Y.T.); yatabe@nbelab.ed.kyushu-u.ac.jp (R.Y.); 3 Intelligent Sensor Technology, Inc., 5-1-1 Onna, Atsugi-shi, Kanagawa 243-0032, Japan; ikezaki.hidekazu@insent.co.jp

**Keywords:** taste sensor, bitterness sensor, response deterioration, quinine hydrochloride

## Abstract

This paper reports the improvement of a bitterness sensor based on a lipid polymer membrane consisting of phosphoric acid di-n-decyl ester (PADE) as a lipid and bis(1-butylpentyl) adipate (BBPA) and tributyl o-acetylcitrate (TBAC) as plasticizers. Although the commercialized bitterness sensor (BT0) has high sensitivity and selectivity to the bitterness of medicines, the sensor response gradually decreases to almost zero after two years at room temperature and humidity in a laboratory. To reveal the reason for the deterioration of the response, we investigated sensor membranes by measuring the membrane potential, contact angle, and adsorption amount, as well as by performing gas chromatography-mass spectrometry (GC-MS), liquid chromatography-tandem mass spectrometry (LC-MS/MS). We found that the change in the surface charge density caused by the hydrolysis of TBAC led to the deterioration of the response. The acidic environment generated by PADE promoted TBAC hydrolysis. Finally, we succeeded in fabricating a new membrane for sensing the bitterness of medicines with higher durability and sensitivity by adjusting the proportions of the lipid and plasticizers.

## 1. Introduction

It is sometimes said that good medicine always tastes bitter. This concept has been reversed with the development of several bitterness-masking techniques. With the quality of life (QOL) of patients attracting increasing attention, especially for pediatric and geriatric patients [1], before marketing an oral bitter medicine, panelists have to take the medicine and evaluate its taste intensity. However, such tests have problems, such as low objectivity and reproducibility, as well as the potential impact of side effects from the medicine. Therefore, objective methods of bitterness evaluation are important for the pharmaceutical industry [2].

The research on taste sensing started in the mid-1990s, before the elucidation of the principle of vertebrate taste receptors [3,4,5]. Since then, many studies on electronic tongues [6,7,8,9,10,11,12] and bioelectronic tongues [13,14] have been carried out for taste assessment. The TS-5000Z taste sensor (Intelligent Sensor Technology Inc., Kanagawa, Japan) is a type of electronic tongue. It is equipped with several sensor electrodes to measure all five basic taste qualities, as well as astringency. Each sensor electrode has a lipid polymer membrane. In accordance with the physicochemical properties of taste qualities, each sensor membrane is designed using different types and amounts of lipids and plasticizers. The lipids are mainly used to adjust the density of electric charges on the surface of the membrane while the plasticizers are added to form a membrane with flexibility. Both lipids and plasticizers affect the hydrophobicity of the membrane [4,15,16,17]. The taste sensor using the lipid polymer membrane has a unique characteristic called ‘global selectivity’, which means that each sensor does not distinguish each chemical substance, but distinguishes taste quality and taste intensity [18,19]. Therefore, this type of taste sensor has the advantage of better selectivity than other electronic tongues, as well as better sensitivity and durability than bioelectronic tongues in bitterness evaluation. In our previous studies, the taste sensor was applied to the objective evaluation of the taste of food and pharmaceutical products (e.g., beer, coffee, traditional Chinese medicines) [19,20,21].

A BT0 sensor is the sensor electrode of the taste sensor used for the bitterness quantification of medicines. The membrane of the BT0 sensor is composed of phosphoric acid di-n-decyl ester (PADE) as the lipid, bis(1-butylpentyl) adipate (BBPA) and tributyl o-acetylcitrate (TBAC) as the plasticizers [22]. In taste solutions, PADE produces negative charges on the surface of the membrane owing to the ionization of phosphate group, while BBPA and TBAC improve the membrane by increasing its flexibility and hydrophobicity. The composition ratios of PADE, BBPA, and TBAC affect the sensitivity and selectivity to bitter substances [22]. The BT0 sensor possesses good sensitivity and high bitterness sensory correlation to the bitterness of medicines, such as quinine hydrochloride, hydroxylammonium chloride, bromhexine hydrochloride, and so forth [19,23]. In addition, the sensor can evaluate the bitterness-masking effect caused by physical and biochemical masking materials [19]. In our previous study, we also provided an effective method of evaluating the bitterness suppression effect of high-potency sweeteners (functional masking materials) by using electrodes for sensing medicine bitterness and high-potency sweeteners [24,25,26,27,28].

Although the BT0 sensor has been used in the pharmaceutical sector, maintaining its long-term performance has become an unresolved issue for practical use. The sensor response gradually decreases with increasing storage time and decreases by half after one year, finally becoming almost zero after two years at room temperature and humidity in a laboratory. This problem not only affects the performance of the BT0 sensor, but also increases the cost of transportation and preservation.

The objectives of this study are to clarify the decrease in the response of the BT0 sensor and to improve the sensor durability to extend its lifetime. In this study, we developed an accelerated deterioration process to reproduce a BT0 sensor after long-term deterioration in a shorter time than by natural deterioration. In addition, we found that the deterioration of the response was caused by the change in the surface charge density due to the hydrolysis of TBAC. We also demonstrated that increasing PADE greatly accelerates the deterioration of the response by creating an acidic environment that induces TBAC hydrolysis. Finally, a new membrane for sensing the bitterness of medicines that possesses higher durability and sensitivity was developed by adjusting the proportions of the lipid and plasticizers.

## 2. Experiment

### 2.1. Materials

**Figure 1:**
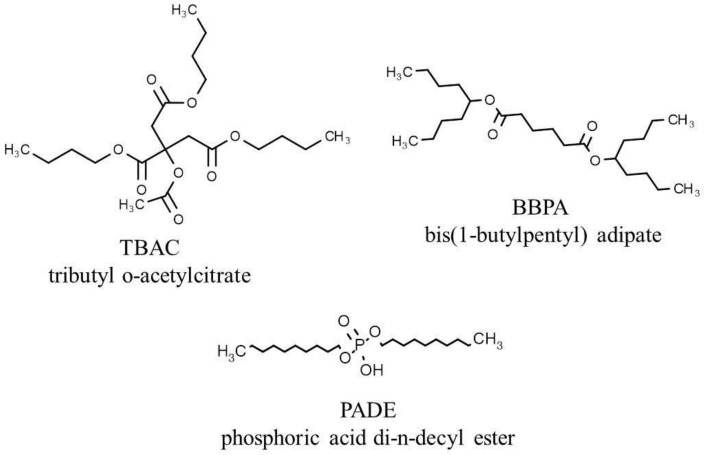
Chemical structures of PADE, TBAC, and BBPA.

Bis(1-butylpentyl) adipate (BBPA) was purchased from Sigma-Aldrich, Inc. (St. Louis, MO, USA). Tributyl o-acetylcitrate (TBAC) and phosphoric acid di-n-decyl ester (PADE) were purchased from Tokyo Chemical Industry Co., Ltd. (Tokyo, Japan). Polyvinyl chloride (PVC), obtained from Wako Pure Chemical, Ltd. (Osaka, Japan), was used as the supporting material. The chemical structures of PADE, TBAC, and BBPA are shown in Figure 1. Tetrahydrofuran (THF) purchased from Sigma-Aldrich was used as the organic solvent.

### 2.2. Fabrication of Sensor Electrode

**Figure 2:**
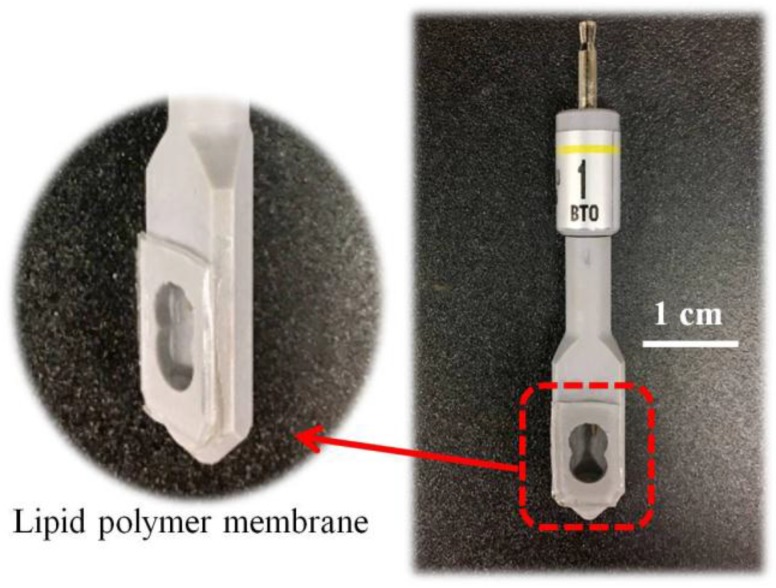
Bitterness sensor electrode (BT0). The sensor electrode is composed of Ag/AgCl electrode and the sensor probe with a lipid polymer membrane.

Lipid polymer membranes with different compositions were fabricated. The fabrication steps were as follows: Firstly, PADE, BBPA, TBAC, the concentrations of which will be detailed in Section 2.5, and 800 mg of PVC were dissolved in 10 mL THF and stirred for one hour. Secondly, the resulting solution was poured into a 90 mm glass Petri dish to dry for three days in a draft chamber. Thirdly, the membrane was cut into pieces of 1 × 1 cm and attached to a sensor probe using an adhesive of 10 mL THF and 800 mg PVC. Fourthly, the sensor probe was filled with 0.2 mL of a solution of 3.3 M KCl and saturated AgCl. Finally, the sensor electrode was completed by fixing an Ag/AgCl electrode to the sensor probe. Figure 2 shows the bitterness sensor electrode. The thickness of the lipid polymer membrane is about 0.73 mm.

### 2.3. Measurement System

The sensor electrode and a reference electrode (Ag/AgCl electrode containing a solution of 3.3 M KCl and saturated AgCl) were used in the measurement. First, the bottom of the sensor electrode with the membrane and the reference electrode were immersed in a reference solution comprising 30 mM KCl and 0.3 mM tartaric acid to obtain a reference potential *V*_r_. The reference solution acted as an alternative to human saliva, and has almost no taste. Second, *V*_s_ was obtained by placing the electrodes in a sample solution. Third, a different potential *V*_r’_ was obtained after the two electrodes were lightly rinsed with the reference solution. We defined the potential difference between *V*_s_ and *V*_r_ as the relative value and the potential difference between *V*_r’_ and *V*_r_ as the CPA (the change in the membrane potential caused by adsorption) value [29,30]. In this paper, all the sensor responses are given as the CPA values. Finally, the CPA value was returned to *V*_r_ by rinsing with a cleaning solution consisting of 30 vol % ethanol and 100 mM HCl. In this study, 0.1 mM quinine hydrochloride was used as a standard solution of a bitter medicine.

### 2.4. Accelerated Deterioration Process

The reason for the deterioration in the response was investigated by comparing the characteristics of the BT0 sensor before and after the deterioration process. However, it is inefficient to wait for the BT0 sensor to degrade naturally. Therefore, we developed an accelerated deterioration process to reproduce a BT0 sensor after long-term deterioration in a shorter time than by natural deterioration. To find suitable conditions for accelerating the deterioration, we placed the sensor membrane in a chamber with constant temperature and humidity (YAMATO IG421, Tokyo, Japan) for 14 and 28 days. The sensor membrane was placed in a glass Petri dish without a cover during the accelerated deterioration process. The storage temperature was set to 45–80 °C. The relative humidity (RH) was set to 20% (regarded as a dry condition), 40%, 60%, or 95% RH. After the accelerated deterioration process, sensor electrodes were fabricated using the deteriorated membranes and used to measure 0.1 mM quinine hydrochloride solution.

### 2.5. Effect of Membrane Components on the Deterioration Rate

To determine the main component causing the response deterioration, the amounts of the lipid and the two plasticizers in the membrane were changed to observe the effect on the sensor response. The deterioration rate *D* was calculated using the following equation:

(1) $$D=\frac{\left|R_{\mathrm{after}}-R_{\mathrm{before}}\right|}{R_{\mathrm{before}}},$$

where $R_{\mathrm{after}}$ is the sensor response after the accelerated deterioration process and $R_{\mathrm{before}}$ is the sensor response before the accelerated deterioration process.

We adopted the control variate method to arrange the sample concentrations. Nine membranes comprising 100% BBPA, 100% TBAC, and 33–278% PADE; five membranes comprising 100% PADE, 100% TBAC, and 33–200% BBPA; and six membranes comprising 100% PADE, 100% BBPA, and 3.3–100% TBAC were made. Here, 100% means that the absolute amount is the same as that in the BT0 sensor. After the accelerated deterioration process, we examined the responses of the sensors using these 20 membranes by measuring 0.1 mM quinine hydrochloride.

### 2.6. Quantitative Analysis by Liquid Chromatography-Tandem Mass Spectrometry

Liquid chromatography–tandem mass spectrometry (LC-MS/MS) was adopted to compare the difference in the amounts of PADE, TBAC, and BBPA in the membrane before and after four-week accelerated deterioration. The sample solutions were prepared as follows. Firstly, 0.2 g of the sensor membrane was dissolved in a 100 mL screw tube. Secondly, the polymer was precipitated by slowly adding 100 mL of acetonitrile. Thirdly, some of the prepared solution was diluted with acetonitrile (diluted 20-fold for PADE quantification or 1000-fold for BBPA and TBAC quantification). Finally, the diluted solution was filtered through a PTFE filter and subjected to LC-MS/MS measurement. The conditions are summarized in Table 1.

### 2.7. Component Detection by Gas Chromatography-Mass Spectrometry

To elucidate the physicochemical reaction occurring during the accelerated deterioration process, we compared the components contained in the BT0 membrane before and after the deterioration test. The membrane components were extracted by solid-phase micro-extraction (SPME) and analyzed by gas chromatography-mass spectrometry (GC-MS). By using the SPME/GS-MS method, the lipid polymer membrane can be measured directly without any solvent. The device used for GC-MS was a SHIMADZU QP2010 (Kyoto, Japan), in which electron ionization (EI) was performed at 70 eV. The column used for all analyses was a Stabilwax^®^-MS, in which the column was 30 m length, 0.25 mm I.D., 0.25 µm film thickness. The oven temperature was initially held at 40 °C for 1 min, after which the temperature was increased to 270 °C at a rate of 10 °C/min. This temperature, 270 °C, was maintained for 4 min. The split ratio was 1:25. The temperature of the inlet was set to 270 °C and the interface temperature was set to 280 °C. An 85 µm polyacrylate film fiber was used to extract the components in the membrane. The samples were cutoffs of BT0 membranes with a weight of 20 mg before and after the accelerated deterioration process.

### 2.8. Measurement of the Amount of Adsorbed Quinine Hydrochloride

In order to determine whether the deterioration in the response was caused by the decrease in the adsorption of quinine hydrochloride, we measured the amount of quinine hydrochloride adsorbed on the membrane using an ultraviolet-visible spectrophotometer (UV-1800, Shimadzu Corporation, Kyoto, Japan). First, the relationship between the concentration and absorbance (calibration curve) was obtained by measuring the absorbance of a known concentration of quinine hydrochloride solution. Five milliliters of 0.1 mM quinine hydrochloride solution was added dropwise onto the surface of a membrane in a glass Petri dish. After allowing the quinine hydrochloride molecules to be adsorbed on the membrane for 30 s, and 3 mL of the quinine hydrochloride solution was removed from the glass Petri dish to measure its absorbance. By measuring the absorbance of the measured solution and the calibration curve, the concentration of the measured solution was calculated. We defined the difference between the amounts of the originally-added quinine hydrochloride solution and the solution after the 30 s adsorption process as the total amount of adsorbed quinine hydrochloride. By dividing by the area of the glass Petri dish, we obtained the amount of quinine hydrochloride adsorbed per square centimeter [30,31].

### 2.9. Measurement of Surface Contact Angle

To investigate the hydrophobicity of the membrane surface, 11 membranes were made for measurement comprising fixed amounts of BBPA and TBAC and 33–278% PADE. By measuring the contact angle of the membrane surface, we determined the change in the hydrophobicity of the membrane surface. The contact angle was measured by a DM500 contact angle meter (Kyowa Interface Science Co., Ltd., Saitama, Japan). In the measurement, a 2 μL water drop was placed on the membrane surface.

### 2.10. Fabrication of a Durable Bitterness Sensor

We optimized the membrane composition to increase the durability (no decrease in response after accelerated deterioration process). To confirm the selectivity of the improved bitterness sensor to the basic taste qualities, sensor electrodes with the new membrane composition were fabricated to measure saltiness, sourness, umami, bitterness, and sweetness. Iso-alpha acid was used to examine the response to acidic bitter materials (bitterness (−)) [32,33], while quinine hydrochloride was used to examine hydrochloride salts (bitterness (+)) [34]. In addition, there are three requirements for the performance of the newly improved bitterness sensor for medicine: selectivity, durability, and concentration dependence. First, the CPA value for standard quinine hydrochloride solution should be at least 30 mV and not be affected by any other basic tastes. Second, the improved sensor should show dependence of the response of the improved bitterness sensor on quinine hydrochloride concentration. Third, the sensor response should remain almost unchanged after accelerated deterioration.

## 3. Results and Discussion

### 3.1. Conditions of the Accelerated Deterioration Process

**Figure 3:**
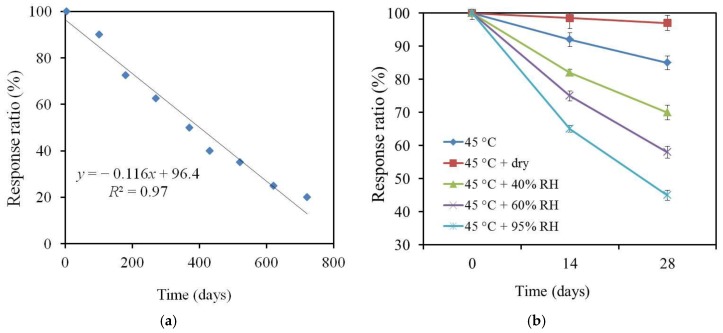
The relationship between the response ratio and preservation time (**a**) at room temperature and humidity in a laboratory and (**b**) at 45 °C with different levels of humidity.

As shown in Figure 3a, the sensor response ratio gradually decreased during the natural deterioration process at room temperature (25 °C) and humidity in a laboratory (uncontrolled, annual average RH of 67% at Kanagawa, Japan). The sensor response dropped by half after 400 days and was zero after 830 days. Although a higher temperature caused a faster deterioration, we used a temperature of 45 °C to examine the effect of the humidity rather than a higher temperature to avoid the loss of membrane components due to evaporation. In Figure 3b, the CPA value remained almost unchanged at 45 °C and 20% RH (dry condition). On the other hand, it decreased under wet conditions. The higher the humidity, the faster the decrease in the CPA value. This result shows that humidity is a factor causing the deterioration in response. In the case of natural deterioration, the response ratio decreased to about 45% after 443 days. In the case of the accelerated deterioration process, the response ratio reached 45% at 45 °C and 95% RH after 28 days, i.e., the deterioration process was about 16 times faster than natural deterioration. In this case, the membrane after one month of accelerated deterioration can produce the same response characteristics as those after one year of natural deterioration. In the following experiment, a deteriorated BT0 membrane was obtained by placing a BT0 membrane on a Petri dish without a cover under the conditions of 45 °C and 95% RH for 28 days.

### 3.2. Relationship between Deterioration Rate and Lipid Mass Ratio

**Figure 4:**
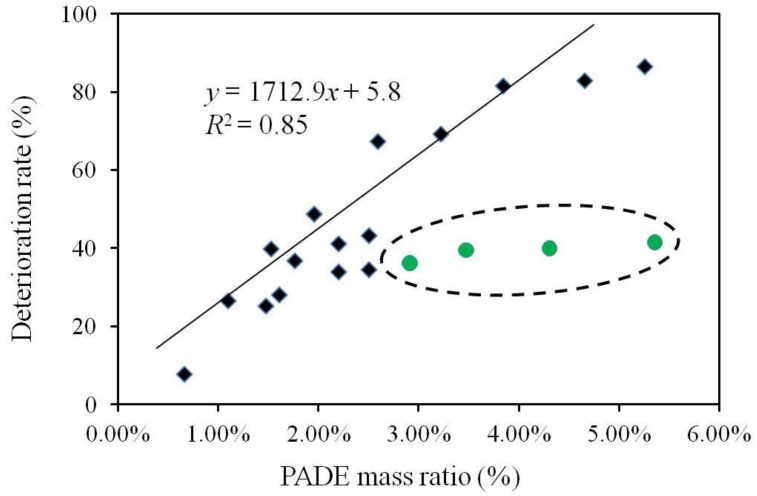
The relationship between deterioration rate and lipid mass ratio. The circled green points represent the sensor membranes with the TBAC mass ratio less than 49%. The regression equation and *R*^2^ were calculated without the green points.

Figure 4 illustrates the deterioration rate defined by Equation (1) when the PADE mass ratio increased from 0.66% to 5.36%. The mass ratios of PADE, TBAC, and BBPA in the BT0 sensor were 1.96%, 65.36%, and 32.68%, respectively. The circled green points represent the sensor membranes with the TBAC mass ratio less than 49%. Excluding the circled green points, the deterioration rate has a strong correlation with the PADE mass ratio (*R*^2^ = 0.85). This finding may indicate that (1) the deterioration rate of BT0 was proportional to the PADE mass ratio when the mass ratio of TBAC exceeded 49%; (2) when the TBAC mass ratio in the membrane was less than 49%, the response deterioration was suppressed.

### 3.3. LC-MS/MS Analysis Results

**Figure 5:**
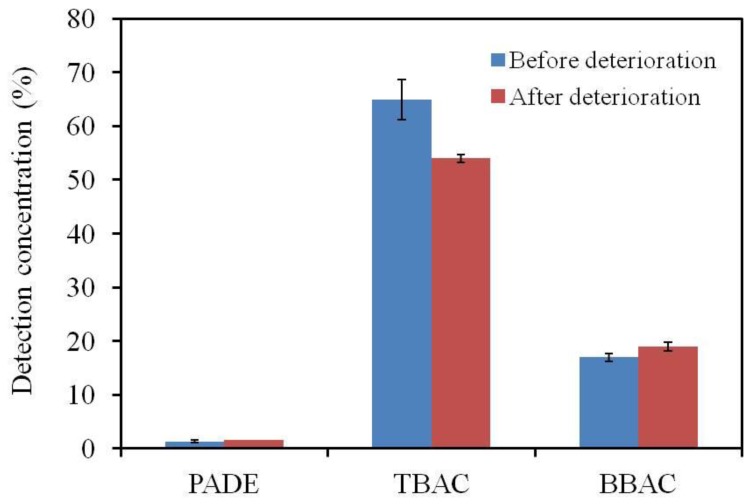
Quantitative comparison of the main components obtained by LC-MS/MS. The results are expressed as the mean ± SD (*n* = 3).

Figure 5 shows that the amount of TBAC was significantly reduced as a result of deterioration. The amounts of PADE and BBPA did not change during the deterioration process, even though PADE had a strong effect on the deterioration rate. This indicates that PADE promoted the process causing the deterioration in the response while remaining unchanged.

### 3.4. GC-MS Analysis of the Membrane Components

**Figure 6:**
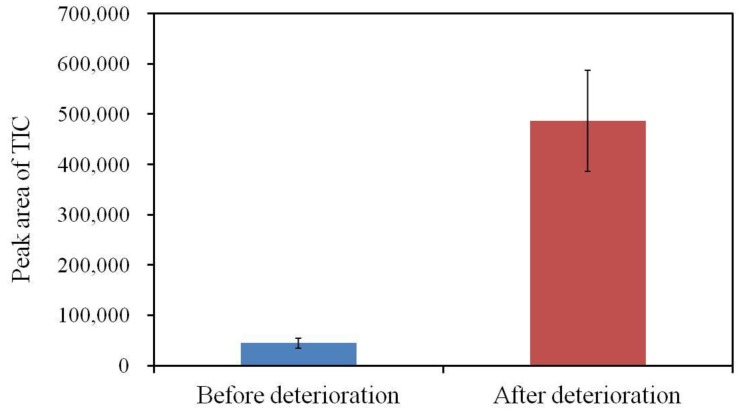
Change in the amount of butyl citrate as a result of the accelerated deterioration process measured by GC–MS. The results are expressed as the mean ± SD (*n* = 3).

Butyl citrate was detected as a degradation product of the plasticizer TBAC. The amount of butyl citrate increased as a result of deterioration (Figure 6), indicating that some TBAC molecules were hydrolyzed during the deterioration process.

### 3.5. Amount of Adsorbed Quinine Hydrochloride

**Figure 7:**
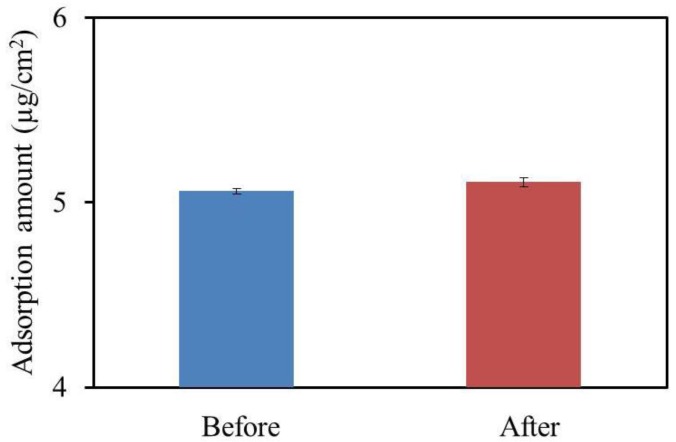
Amounts of adsorbed quinine hydrochloride before and after the accelerated deterioration process. The results are expressed as the mean ± SD (*n* = 3).

Figure 7 shows that the same amount of quinine hydrochloride was adsorbed before and after the deterioration. As we reported in previous studies, the sensor response was affected by both the surface charge density and the amount of adsorption [15,35]. Therefore, the result indicates that the change in the surface charge density of the BT0 membrane during the accelerated deterioration process probably caused the decrease in the sensor response.

### 3.6. Reference Potential and Contact Angle of Lipid Polymer Membrane

**Figure 8:**
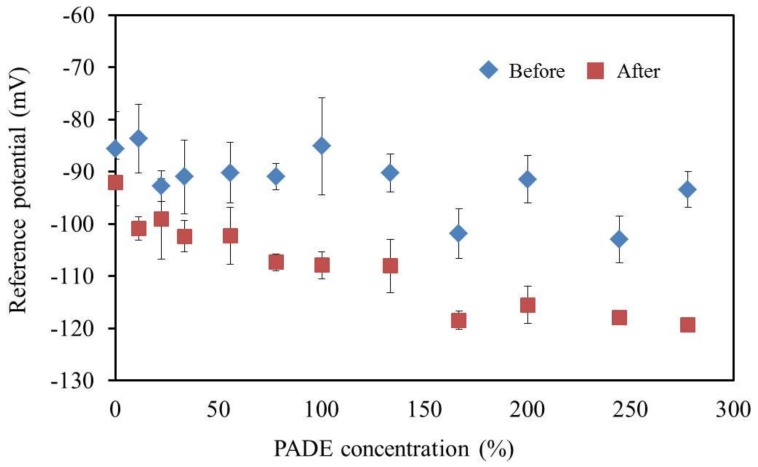
The relationship between the reference potential and lipid concentration. The results are expressed as the mean ± SD (*n* = 4).

The reference potential (*V*_r_) was significantly lower after the accelerated deterioration process, which indicates that negatively-changed substances were generated, which increased the surface charge density (Figure 8).

**Figure 9:**
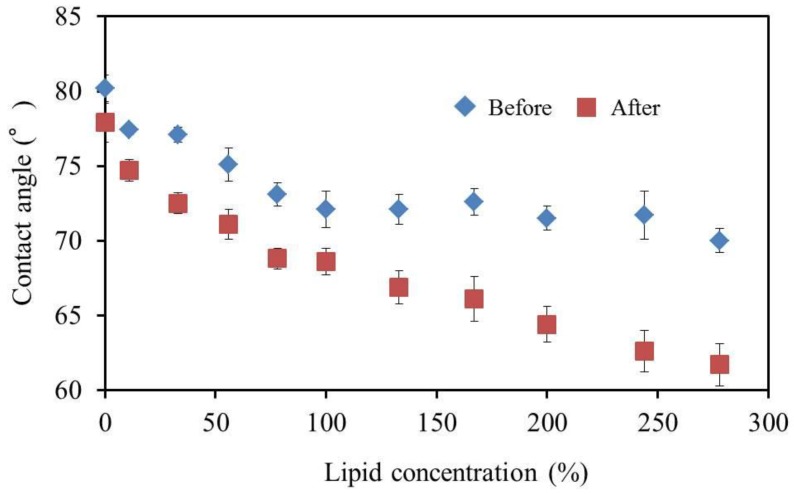
The relationship between the surface contact angle and lipid concentration. The results are expressed as the mean ± SD (*n* = 4).

The contact angle decreased as a result of deterioration (Figure 9). This result indicates that the surface became more hydrophilic during the deterioration process. During the deterioration process, TBAC was hydrolyzed into butyl citrate and acetic acid, which caused the membrane surface to become hydrophilic and increased the negativity of the reference potential. The role of PADE during the deterioration was to create an acidic condition in the membrane allowing TBAC to hydrolyze, because PADE is a phosphate ester. In addition, the PADE content affects the deterioration rate more than the TBAC content. Therefore, it is considered that the reduction in the PADE content improves the durability of the sensor.

### 3.7. BT0 Sensor with Improved Durability and Sensitivity

**Figure 10:**
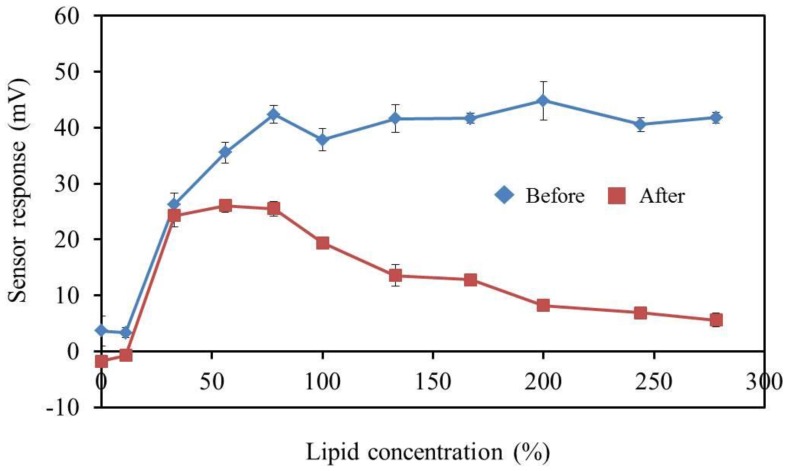
The relationship between the sensor response and lipid concentration. The results are expressed as the mean ± SD (*n* = 4).

**Figure 11:**
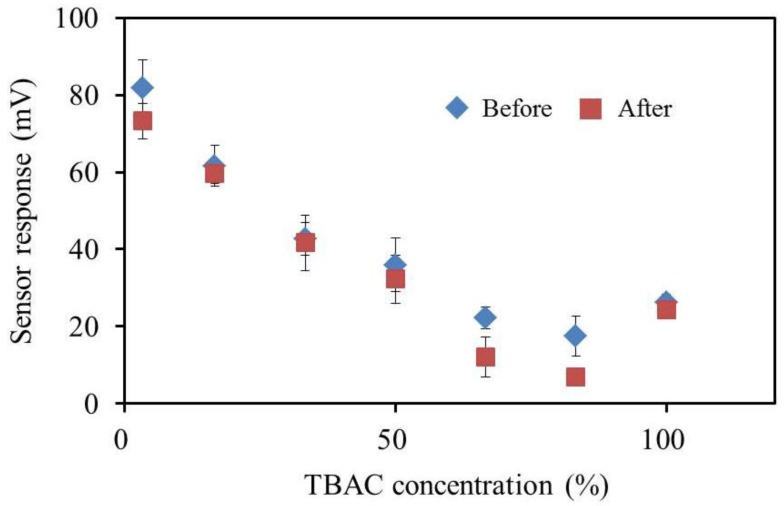
The relationship between the sensor response and TBAC concentration. The results are expressed as the mean ± SD (*n* = 4).

As shown in Figure 10, we found that the deterioration rate of the CPA value was reasonably low when 33% or less PADE was added. This membrane might be considered as relatively durable because of the relatively weak acidic environment. However, the CPA value was about 25 mV, only 70% of that of the conventional BT0 sensor (about 36 mV to 0.1 mM quinine hydrochloride) and did not satisfy the required response. On the other hand, the CPA value increased when the amount of the plasticizer TBAC included in the BT0 membrane was decreased. However, TBAC is considered to be necessary to match the results of bitterness sensory tests for various medicines in the sensor development stage [22]. As shown in Figure 11, the responses of sensor electrodes with 17%, 34%, and 50% TBAC exhibited no significant difference before and after the accelerated deterioration, indicating high durability, as well as a sufficient response to quinine hydrochloride comparable to that of the conventional BT0 sensor [19]. Therefore, the new sensor is not expected to deteriorate during one year of storage at room temperature and humidity in a laboratory.

**Figure 12:**
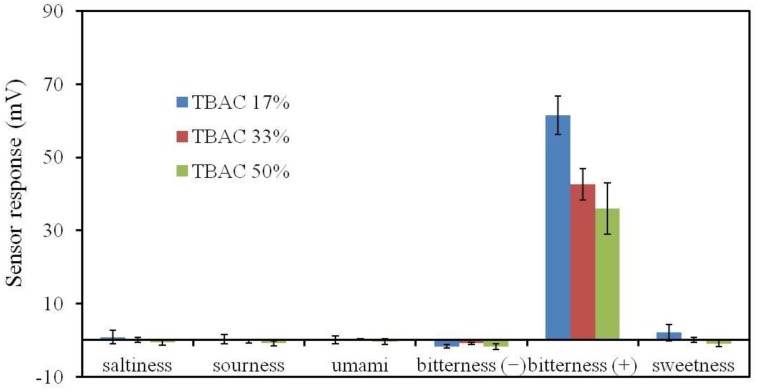
The sensor response to five basic tastes. The reference solution (RS) comprised 30 mM KCl, and 0.3 mM tartaric acid; the saltiness sample comprised 300 mM KCl, and 0.3 mM tartaric acid; the sourness sample comprised 30 mM KCl, and 3 mM tartaric acid; the umami sample comprised 10 mM sodium glutamate and RS; the bitterness (+) sample comprised 0.1 mM quinine hydrochloride and RS; the bitterness (−) sample comprised 0.01 vol % iso-alpha acid and RS; and the sweetness sample comprised 1 M sucrose and RS.

**Figure 13:**
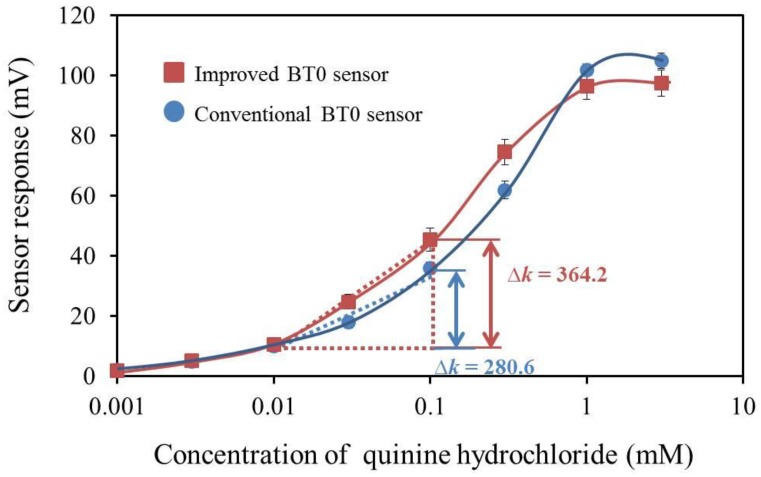
Dependence of the response of the improved bitterness sensor and conventional bitterness sensor on quinine hydrochloride concentration.

As shown in Figure 12, all three sensors showed good sensitivity to bitterness (+) and met the response requirement of over 30 mV to 0.1 mM quinine hydrochloride. In addition, the sensor did not respond to any other basic tastes, showing good selectivity to bitterness (+). From the viewpoint of sensitivity, the optimized membrane composition is 33% PADE, 100% BBPA, and 17% TBAC. Therefore, we measured the concentration dependence of this sensor for quinine hydrochloride. Figure 13 shows that the sensor response of the improved sensor was proportional to the logarithm of the concentration of quinine hydrochloride, which expresses the bitterness intensity, in accordance with the Weber-Fechner law [36]. The improved sensor shows the same linear response range as the conventional BT0 sensor of between 0.01 and 1 mM quinine hydrochloride. Since the bitterness intensity corresponding to between 0.01 and 0.1 mM quinine hydrochloride is the common range for sensory scores felt by humans, we chose this range to compare the sensor sensitivity. The improved sensor has a higher response-concentration slope than the conventional sensor, which indicates that the improved sensor has higher sensitivity in this bitterness range.

## 4. Conclusions

We investigated the reason for the deterioration in the response of the BT0 sensor and fabricated a new lipid polymer membrane for bitterness with improved durability and sensitivity. We concluded that the proximate cause for the deterioration in the response of the BT0 sensor is the increase in the surface charge density. Experimental results showed that TBAC was involved in hydrolysis and produced negatively-charged substances, which increased the surface charge density. It was also revealed that the rate of deterioration is strongly promoted by increasing the mass ratio of PADE because the acidic environment generated by PADE promotes TBAC hydrolysis. In addition, a low ratio of TBAC can both suppress the deterioration rate and improve the sensitivity of the sensor. Therefore, the results motivated us to improve the sensor by reducing the amounts of PADE and TBAC in the membrane. In conclusion, we produced a new sensor membrane consisting of 33% PADE, 100% BBPA, and 17% TBAC. This sensor showed high durability and sensitivity compared with the conventional BT0 sensor.

The next step is to measure commercially available medicines to confirm the correlation between the sensor response and the bitterness sensory score.

## Figures and Tables

**Table 1:** LC-MS/MS conditions.

LC instrument	Shimadzu LC-20A (Kyoto, Japan)
LC column	Cadenza CD-C18 (2.0 × 100 mm, 3 µm, Portland, OR, USA)
Column temperature	50 °C
Mobile phase	A: 10 mmol/L Ammonium acetate/H_2_O
	B: Acetonitrile
Flow rate	0.3 mL/min
Gradient conditions	<PADE>
	0.0 min → 10.0 min: B20% → B90%
	10.0 min → 12.0 min: B90%
	12.1 min → 20.0 min: B20%
	<BBPA/TBAC>
	0.0 min → 5.0 min: B60% → B95%
	5.0 min → 15.0 min: B95%
	15.1 min → 20.0 min: B60%
Injection volume	1 µL
MS instrument	API 4000 (AB SCIEX)
Ionization	ESI
Polarity	<PADE> negative; <TBAC/BBPA> positive
Scan type	SRM
	<PADE> Q1: m/z 377.3 → Q3: m/z 237.1
	<BBPA> Q1: m/z 399.3 → Q3: m/z 273.2
	<TBAC> Q1: m/z 403.3 → Q3: m/z 329.3
